# Extremity compartment syndrome: A review with a focus on non-invasive methods of diagnosis

**DOI:** 10.3389/fbioe.2022.801586

**Published:** 2022-07-18

**Authors:** Martin Novak, Marek Penhaker, Pavel Raska, Leopold Pleva, Martin Schmidt

**Affiliations:** ^1^ Trauma Surgery Clinic, University Hospital Ostrava, Ostrava, Czechia; ^2^ Department of Cybernetics and Biomedical Engineering, Faculty of Electrical Engineering and Computer Science, VSB—Technical University of Ostrava, Ostrava, Czechia; ^3^ Department of Occupational and Process Safety, Faculty of Safety Engineering, VSB—Technical University of Ostrava, Ostrava, Czechia

**Keywords:** acute compartment syndrome, continuous measurement, detection, non-invasive diagnosis, bioimpedance measurement

## Abstract

The article deals with an overview of acute extremity compartment syndrome with a focus on the option of non-invasive detection of the syndrome. Acute extremity compartment syndrome (ECS) is an urgent complication that occurs most often in fractures or high-energy injuries. There is still no reliable method for detecting ECS. The only objective measurement method used in clinical practice is an invasive measurement of intramuscular pressure (IMP). The purpose of this paper is to summarize the current state of research into non-invasive measurement methods that could allow simple and reliable continuous monitoring of patients at risk of developing ECS. Clinical trials are currently underway to verify the suitability of the most studied method, near-infrared spectroscopy (NIRS), which is a method for measuring the local oxygenation of muscle compartments. Less explored methods include the use of ultrasound, ultrasound elastography, bioimpedance measurements, and quantitative tissue hardness measurements. Finding a suitable method for continuous non-invasive monitoring of the syndrome would greatly improve the quality of care for patients at risk. ECS must be diagnosed quickly and accurately to prevent irreversible tissue damage that can occur within hours of syndrome onset and may even warrant amputation if neglected.

## Introduction

The muscles and soft tissues of the limbs are divided into several compartments in every part of an extremity. Compartments are spaces enclosed by the fascia and the skeletal system. Extremity compartments usually contain muscles with similar functions. A compartment syndrome is a condition that can occur due to increased tissue pressure in this closed fascial space, compromising the circulation to the nerves and muscles within the involved compartment (Mubarak et al., 1978).

This work is focused on the current possibilities in diagnosing acute extremity compartment syndrome (ECS). If neglected, this syndrome can cause irreversible limb damage that may even require amputation. Therefore, it is necessary to diagnose this syndrome in the early stages and start treatment as soon as possible (Schmidt, 2017).

### Epidemiology

Up to 75% of ECS cases are caused by a fracture (Shadgan et al., 2008). The most common cause is a fracture of the tibial shaft because of an injury, causing up to 36% of all compartment syndromes. The second most commonly affected part of the body is the forearm with up to 9% of all cases (Pechar and Lyons, 2016). It is often believed that open fractures provide additional space for compartment tissue expansion and thus reduce the risk of ECS. However, studies have shown that there is no difference in intramuscular pressure (IMP) or risk of developing ECS between open and closed fractures (Gourgiotis et al., 2007). The syndrome occurs up to ten times more often in men than in women, probably due to higher muscle mass. The group of patients who are most at risk of developing ECS includes men under the age of 35 with a tibial fracture. Higher risk is also in patients after high-energy injuries with a fracture or soft tissue damage. Not all cases of ECS are caused by injury.

ECS can also have iatrogenic causes, i.e., long-term compression of a limb during lithotomy or Trendelenburg positions (Meyer et al., 2002) (Neagle et al., 1991) (Chung et al., 2010) (Simms and Terry, 2005), or injection of pressurized fluid into the compartment (Seiler et al., 1996). ECS can also arise due to crush syndrome (McQueen et al., 2000), spontaneous bleeding in a hemophiliac (Dumontier et al., 1994), or after intramedullary nailing (Tischenko and Goodman, 1990; Madeja et al., 2022). Repeated muscle exertion can also lead to a chronic version of limb compartment syndrome (Tucker, 2010).

### Pathophysiology

**FIGURE 1:**
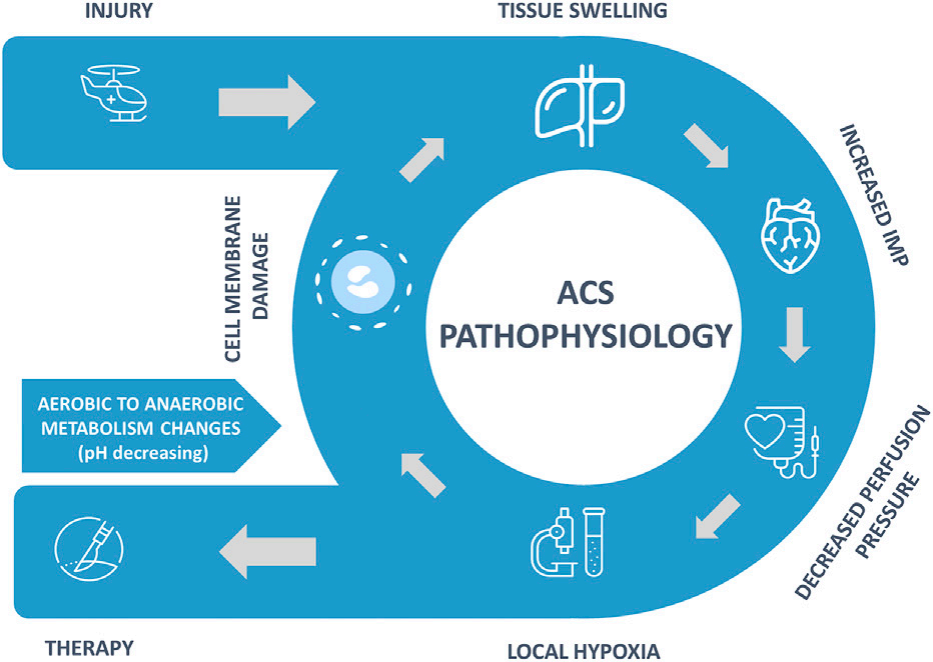
ECS pathophysiology.

The exact pathophysiology mechanism of ECS continues to be debated (McMillan et al., 2019), but a general development of the syndrome is illustrated in Figure 1. The injury results in swelling of intra-compartmental tissues and bleeding into the compartment. Compartment syndromes can arise in any area of the body that has little or no capacity for tissue expansion. When fluid enters a fixed-volume compartment, e.g., from bleeding, both the tissue pressure and venous pressure increase. When these exceed capillary perfusion pressure (CPP), capillary collapse with muscle and nerve ischemia occurs.

A similar reduction occurs in the CPP when the compartment size decreases (e.g., external compression) due to an increase in IMP, as well as a reduction in the arteriolar pressure (Donaldson et al., 2014). Dilation in the arteriole system caused by injury, along with collapsing smaller vessels and increased permeability, leads to increased fluid extravasation and raised interstitial fluid pressure. As it increases, perfusion to tissue becomes decreased, leading to tissue hypoxemia.

The combination of hypoxia, increase in oxidant stress, and development of hypoglycemia in the compartmental tissue causes cell edema due to a shutdown of the ATPase channels that maintain cellular osmotic balance. Early ACS microvascular dysfunction results in a decrease in capillary perfusion and an increase in cellular injury and is associated with a severe acute inflammatory component. The loss of cell-membrane potential results in an influx of chloride ions, leading to cellular swelling and ongoing cellular necrosis. The increase in tissue swelling worsens the hypoxic state and creates an ongoing positive feedback loop.

### Therapy and complications

The cascade of elevated pressure compromises the microcirculation with decreased oxygen and nutrient delivery. This leads to tissue anoxia and eventually to myonecrosis. Systemic changes have been reported as remote changes in liver and kidney function (Mauffrey et al., 2019). The only definitive solution to manifested ECS is a surgical intervention called fasciotomy (Saber, 2014; Schmidt, 2017; Osborn and Schmidt, 2020).

Even though fasciotomy is the definitive solution to early diagnosed ECS, it also has its shortcomings and complications. These include the need for suturing of the wound, cosmetic deformities, pain and nerve injuries, permanent muscle weakness, and chronic venous insufficiency. Delayed fasciotomy may however lead to worse damage in the form of sensory deficits of the limb, claw toes and may even warrant amputation (Velmahos et al., 1997). The condition for successful fasciotomy is its timely execution and is only of benefit during a brief time interval. Permanent damage may already be present at the onset of the symptoms and a reliable method for early ECS detection would be beneficial (Van Den Brand et al., 2004).

In case of late presentation or missed diagnosis of ECS, some authors suggest non-operative management, since surgical decompression can be harmful and of little benefit at that point. It has been demonstrated that fasciotomies performed later than 8 h after diagnosis of ECS were associated with a significantly higher risk of infection (Velmahos et al., 1997). In these situations, case by case evaluation is mandatory (Rothenberg et al., 2019).

With prolonged ischemia, a “no-reflow phenomenon” occurs that is characterized by capillary occlusion from endothelial swelling, clogging of capillaries with red and white blood cells, and increased interstitial pressure. Successful reperfusion liberates the byproducts of muscle ischemia and cell necrosis (rhabdomyolysis) into the circulation, including potassium, phosphate, organic acids, myoglobin, creatine kinase, and thromboplastin. The systemic effects of these byproducts may include hyperkalemia, hyperphosphatemia, metabolic acidosis, and myoglobinuria. These effects can cause acute kidney injury, elevation of serum creatine kinase concentration, and disseminated intravascular coagulation (Modrall and Eidt, 2011).

Reperfusion following fasciotomy causes local and systemic effects that can be life-threatening and can complicate wound management. Increased blood flow in the muscle following the restoration of normal tissue pressure usually causes muscle edema. The extent of extremity swelling depends upon the duration and severity of ischemia, the predominant muscle cell type within a muscle, the location and mass of ischemic muscle, and the status of the venous circulation. Animal studies show that cellular damage starts about 3 h after a complete ischemic insult and is nearly complete by 6 h (Barie and Mullins, 1988). In human beings, the level of tolerance varies and not all ischemic insults are complete. Patients with underlying peripheral artery disease may exhibit smaller swelling due to the protective effects of pre-existing arterial collaterals. Swelling may be limited if there is incomplete reperfusion due to microvascular thrombosis.

There is no validated way to determine whether a muscle affected by compartment syndrome will recover and whether performing a fasciotomy will prevent necrosis of the affected muscles and nerves within the compartment (Schmidt, 2017). Fasciotomy should be performed immediately when ECS diagnosis is confirmed, unless symptoms have been present for more than 8 h, after which it will cause more harm than benefit. It is generally accepted that it is better to perform a fasciotomy that ultimately proves unnecessary than to perform a fasciotomy late in a symptomatic patient and risk the consequences of neglected ECS (Gourgiotis et al., 2007; Taylor et al., 2012). Performing a fasciotomy in the late stages of the syndrome (more than 8 h after onset) is contraindicated, as it will not benefit the patient and significantly increases the risk of infection (Coccolini et al., 2020).

Lower extremity fasciotomy may predispose the patient to deep venous thrombosis (DVT). Skeletal muscles have an important role in forcing blood out of veins and back toward the heart. During exercise, the muscle pumping mechanism provides more than 30% of the energy required for blood circulation. In particular, the calf is recognized as a highly effective venous pump, capable of generating pressures greater than 200 mm Hg. Components of the calf muscle pump include competent venous valves, the pressure generated by the gastrocnemius and soleus muscles, and the enclosing fascia and skin surrounding the calf muscles. Disruption of any of these elements may lead to calf muscle pump dysfunction, which increases venous pressure and can lead to DVT (Bermudez et al., 1998).

Despite numerous articles in the literature regarding fasciotomy, there is little published about the complications of the fasciotomy procedure. Patients with open fasciotomy wounds are at a risk for infection, and incomplete or delayed fasciotomies can lead to permanent nerve damage, loss of limb, multi-system organ failure, rhabdomyolysis, and death. If muscle injury is extensive, either from prolonged ischemia or from a direct crush, large amounts of myoglobin may be released as the muscle is perfused after fasciotomy.

ECS carries a substantial risk of morbidity and mortality, including chronic pain, permanent functional impairment, amputation, and death. Timely surgical intervention with fasciotomies to release the increased pressure is the standard of care but does not guarantee a fully functional limb. A problematic complication is the development of surgical site infection (SSI), which itself can lead to sepsis, amputation, and death. Previous studies have demonstrated that the rate of postoperative infection in cases of ECS treated with decompressive fasciotomy is as high as 30%. Yet, there is a lack of studies examining the factors that may be contributing to this high infection rate. Merchan and colleagues reported on a retrospective series of 142 patients who underwent fasciotomy for compartment syndrome that a factor independently associated with the likelihood of infection was time to closure after fasciotomy (Merchan et al., 2022). Patients who developed infections had a median of 7 days until closure, whereas patients who did not develop an infection had a median of 4 days from fasciotomy to closure. The ability to close is often limited by swelling within the compartments, but the strong correlation between time to closure and infection risk could suggest that it is worth exploring other methods if the wound cannot be closed primarily within the given time frame. Other factors, such as debridement at the time of surgery and the type of closure, were not associated with a change in the risk for infection. This serves us to emphasize that with ECS, when considering the risk of infection, the key factor is not necessarily the type of treatment, but the timing. All surgeons need to have a comprehensive knowledge of the relevant anatomy, and the techniques for performing a proper fasciotomy to minimize the morbidity and mortality associated with failure to adequately treat compartment syndromes (Bowyer, 2015).

## Current process of diagnosing extremity compartment syndrome

Currently, mainly clinical symptoms are used in the diagnosis of ECS. This method requires the patient’s willingness to cooperate. The only objective method used for detection of ECS is currently direct IMP measurement, but physicians usually measure IMP only if symptoms prove to be ambiguous or as a supplementary examination to aid in the diagnosis (Ulmer, 2002). Objective quantifiable measurement method for detection of the early stages of the disease would aid mainly in the diagnosis of patients who are in shock, unconscious, senile, or too young to communicate.

### Clinical signs of extremity compartment syndrome

ECS clinically manifests through several symptoms. Classically the “five Ps” (pain, pallor, pulselessness, paralysis, and paresthesia) are thought of as the symptoms heralding a compartment syndrome (Donaldson et al., 2014). Swelling and palpable tenseness over a muscle compartment are the first signs of a compartment syndrome and are manifestations of increased pressure within the compartment. However, these signs are only crude indications of increased IMP, and other physical findings must also be sought (Mubarak et al., 1978).

The pain is often described as burning, deep in nature, and is reproduced with passive stretching of the muscles in the compartment. Pulselessness and paralysis are rare, only occurring after an arterial injury or after a substantial amount of time has elapsed (Whitesides and Heckman, 1996). Physical signs are often few but there may be a firm, wooden feeling on deep palpation. Reduced two-point discrimination or vibration sense may be found in the early stages. If a major sensory deficit is evident the syndrome is already far advanced (Donaldson et al., 2014).

Thus, by combining the palpation test and checking for the presence of several of these clinical signs, high specificity but only low sensitivity can be achieved. In addition, the patient must cooperate in establishing the diagnosis. Pain can also be lessened by peripheral nerve blockade that can be used in cases of orthopedic trauma and can also mask the presence of ECS (Tran et al., 2020).

### Intramuscular pressure measurement

When diagnosing ECS, in many cases an objective measurement method would be beneficial in making the diagnosis. The main disadvantage of direct IMP measurement is its invasive nature and its associated complications. It can also be influenced by subjective measurement error (Large et al., 2015) because each physician can use a slightly different approach to the measurement, for example, depth of measurement site (Nakhostine et al., 1993) and the location of measurement site (Heckman et al., 1994) can influence the results.

The physiologic value of IMP is up to 8 mm Hg at rest, slightly higher in children, up to 16 mm Hg (McQueen and Duckworth, 2014). Measurement of IMP provides an objective assessment that can aid in the diagnosis of compartment syndrome. It is most often used in patients unable to provide feedback to the physician. Many authors recommend measuring IMP in all patients with fractures who fall into the risk group or are suspected of developing ECS. With the help of IMP measurements, it is possible to detect the developing ECS before the onset of symptoms, so the time spent waiting for the development of symptoms to confirm the diagnosis can be avoided. This enables early intervention and greatly improves the prognosis of the disease (Shadgan et al., 2008).

The thresholds of IMP signaling ECS vary among authors, Mubarak et al. suggesting the threshold of 30 mm Hg (Mubarak et al., 1978), Matsen et al. 45 mm Hg (Matsen et al., 1980). The value of IMP should however always be compared to the value of blood pressure of the patient. Perfusion pressure is defined as the difference between diastolic blood pressure and IMP. Decrease of perfusion pressure under 30 mm Hg is usually used as an indicator that ECS may be present.

Perfusion pressure criteria have a high negative predictive value, so they are better at ruling out ECS rather than confirming the diagnosis. Determining the presence of ECS is slightly inaccurate for this method. Studies indicate that even if measured perfusion pressure values are low enough to classify for ECS diagnosis, compartment syndrome might not be present in some cases (Harris et al., 2006; Schmidt et al., 2020). Thus, it is possible that patients have low perfusion pressure and still do not need fasciotomy.

IMP measurement is an accurate method that should be one of the factors contributing to the decision whether to perform a fasciotomy, but even this method is not infallible. IMP may vary among compartments, as shown in both healthy and fractured legs in children, where the anterior compartment showed higher values of IMP than other compartments (Bussell et al., 2019). In patients with bone fractures, IMP also depends on the distance of the measurement site from the fracture. Peak IMP is found within 5 cm of the fracture (Heckman et al., 1994). The exact value of IMP also varies depending on the measurement technique. There is a difference between the measured values when measuring with an infusion needle, side-ported needle, or a slit or wick catheter. The classic infusion needle was shown to be less precise than the wick catheter (Mubarak et al., 1978). The slit and wick catheters were shown to measure similar values (Shakespeare et al., 1982) and unlike needles can be used for continuous monitoring for up to 24 h.

### Other invasive methods of extremity compartment syndrome detection

Even though IMP measurement is the only method available in clinical practice, there have been studies concerning other methods of detecting the syndrome invasively. Two of the methods use localized analysis of metabolic changes in the injured muscle compartment, glucose monitoring, and pH monitoring. Glucose monitoring in the muscle was investigated in a canine model, where it showed 100% sensitivity and 75% specificity, and it enabled quick monitoring of changes in metabolic activity. The technology of the sensors would however need to be improved for human trials (Doro et al., 2014). Intramuscular measurement of pH has been enabled only recently through the development of pH probes. Initial studies show the potential of this method to deliver more accurate and faster results of monitoring the onset of ECS (Johnstone et al., 2013). In a prospective clinical study on 39 patients suffering from ECS, intramuscular monitoring of pH performed better than IMP measurement, with an identified critical pH value of 6.38 with sensitivity of 95% and specificity of 80% in ECS detection (Elliott, 2007).

## Non-invasive diagnostic methods

Many research studies aimed to find a method that would allow long-term continuous monitoring of the onset of ECS. Because it is not possible to measure IMP non-invasively, other methods have been evaluated that can measure different phenomena that can be correlated with IMP values or provide different information about the tissues in the compartment that are also compromised in ECS, like the degree of oxygenation. The most researched methods include quantitative measurement of tissue hardness, which is basically a quantified palpation examination, measurement of local oxygenation by reflection of radiation in the near part of the infrared spectrum, and the use of methods using ultrasound. Less investigated methods include magnetic resonance imaging, scintigraphy, laser doppler flowmetry, and bioimpedance. Table 1 summarizes the investigated methods for non-invasive ECS detection.

**TABLE 1:** Comparison of available diagnostic methods.

Method	Invasive	Continuous	Advantages	Disadvantages
IMP (Intramuscular Pressure)	Yes	Yes	Accurate, verified	Painful for the patient, risk of infection
Quantitative Hardness	No	No	Simple use	Low specificity, affected by the amount of subcutaneous fat
NIRS (Near Infrared Spectroscopy)	No	Yes	Simple and continuous monitoring	Depth max. 3 cm, unreliability of data acquisition, requires measurement on the control compartment, affected by pigment, skin damage, hematomas
SE (Strain Elastography)	No	No	Allows imaging of the compartment - it is not affected by hematomas and subcutaneous fat	Has not been validated in patients with ECS
SWE (Shear Wave Elastography)	No	No	Allows direct measurement of tissue stiffness	Not verified in patients with ECS, expensive, poor repeatability of measurements
PPLL (Pulsed Phase-Locked Loop)	No	Yes	Simple and continuous monitoring	Has not been verified in patients with ECS, may be affected by low blood pressure
Bioimpedance	No	Yes	Simple and continuous monitoring	Few studies performed, requiring control contralateral compartment

MRI showed it could be used to identify compartments with manifested ECS, but only at a late stage of the syndrome. In the initial phase, no pathological findings were seen in the images even after administration of contrast medium Gd-DPTA (gadolinium-diethylenetriamine pentaacetic acid) (Rominger et al., 2004). Loss of normal muscle architecture appeared as inhomogeneous bright areas in T2-weighted turbo spin images, further enhanced in brightness by contrast medium. MRI has been used with some success in diagnosing the chronic version of the syndrome, where the authors found a statistically significant increase in T2-weighted signal intensity in the affected compartment that disappeared after fasciotomy (Verleisdonk et al., 2001). These results were further verified and the method seems suitable for diagnosing the chronic version of limb compartment syndrome (Andreisek et al., 2009; Ringler et al., 2013). Dynamic magnetic resonance spectroscopy at 9.4 Tesla was capable of detecting ECS in an animal model (Ohta et al., 2021), but the method is expensive and time-consuming, not allowing continuous monitoring in regular clinical practice. It could however be suitable for the chronic version of the syndrome, where the symptoms exacerbate after exercise and can therefore be planned.

The same disadvantages were encountered with scintigraphy (Edwards et al., 1999). The authors used 300 MBq of technetium isotope (99Tcm-methoxyisobutyl isonitrile). The patients in the study showed regional abnormalities in muscle perfusion in the calf following exercise. This method demonstrated the ability to detect the chronic form of compartment syndrome with a sensitivity of 80% and a specificity of 97% when tested on 46 patients. However, it is not suitable for long-term continuous monitoring of ECS development. For the chronic form of compartment syndrome, one study was also performed using a laser doppler flowmetry to measure the blood flow to the affected muscle compartment (Abraham et al., 1998). However, this method has not been tested for the acute form of the syndrome.

Only a few studies have been published that have used bioimpedance measurement for detection of ECS (Tonkovic et al., 2000). The electrical impedance of tissue depends on its properties, i.e., its type, volume, and blood flow. In clinical practice, bioimpedance measurements are mainly used to estimate body composition. The method uses low amplitude alternating current in the order of tenths of a milliampere at frequencies in the range from 100 Hz to 1 MHz (Khalil et al., 2014).

A study looking at bioimpedance as a means of detecting ECS managed to show a correlation between the symptoms of ECS and the results of bioimpedance measurements in 29 patients with unilateral ECS of the leg. The authors found significant differences in bioimpedance between healthy and damaged limbs. The damaged limb showed a higher IMP and a lower value of bioimpedance. The group with the most apparent clinical manifestation of the syndrome showed on average a difference of 18 mm Hg between a healthy and a damaged limb and a corresponding difference in impedance of 346 Ω. The group with no manifestations of ECS showed a difference of only 1.2 mm Hg and 29.9 Ω (Tonkovic and Voloder, 1998). The disadvantage of the method used is that it is necessary to measure on both limbs and therefore can only be used to assess unilateral ECS. The advantage of the method is that it can be measured easily, continuously, and non-invasively over a long period.

The method of measuring bioimpedance has been barely tested for this purpose but could also be a suitable method for non-invasive monitoring of the onset of ECS. Measurement of muscle bioimpedance in the form of electro impedance myography was studied for the diagnosis of neurological disorders (Rutkove, 2009). Impedance values are influenced by factors such as tissue swelling and myocyte loss. Measurement of bioimpedance also comes to the fore in the diagnosis of lymphedema that is characterized by increased concentrations of extracellular fluid in the limb (Ward, 2009). Impedance spectroscopy showed the ability to detect swelling of one limb when a healthy paired limb was used for control measurement (Watanabe et al., 1998). Bioimpedance was also used to assess muscle damage in injuries (Nescolarde et al., 2013) and rates of muscle contraction (Nescolarde et al., 2013; Bartels et al., 2015).

### Quantitative tissue hardness measurement

When ECS occurs, the affected compartment tissues expand, and the IMP increases. This also affects other tissues of the limb, and this is reflected in the overall hardness of the tissues of the limb. The method of measuring the surface hardness of the limbs uses the analysis of the indentation curve when the measuring device presses on the limb. The curve indicates the depth of indentation corresponding to the force exerted on the limb by the measuring device.

In 1994, Steinberg and Gelberman built a device that would derive IMP by measuring limb tissue hardness (Steinberg and Gelberman, 1994). The device was tested on three dogs and then on 6 patients with indicated compartment syndrome. The correlation coefficient with the IMP measurement in both cases ranged from 0.87 to 0.99. The device was further tested on 75 patients with suspected ECS (Dickson et al., 2003). The results showed the specificity of 82% in ECS detection with the tested device compared to 96% of the invasive IMP measurement method. Therefore, the study does not support the use of a tissue hardness-based instrument for the diagnosis of ECS.

In 2004, a patented second version of the device was created (Steinberg, 2005). Testing of this version was done on a total of 71 limb compartments of 18 healthy patients. Cuff inflation was used to simulate the increased pressure. The measurement results were compared with the results measured by the Stryker pressure monitor (Stryker Surgical; Kalamazoo, Michigan USA). The study achieved a statistically significant correlation coefficient of 0.84 and showed a strong linear dependence of tissue hardness on IMP. The factors that most influenced the measurement results were gender and the amount of subcutaneous fat in limbs. In 2011, the device was extensively tested on 205 limb compartments and the results were compared with IMP measurements. The correlation coefficient of the tissue hardness measurement with the IMP measurement was 0.78 (Steinberg et al., 2011).

Another research group assembled a device for measuring tissue hardness and then tested it on the forearms of 189 children and 20 adult patients (Joseph et al., 2006). The results of the study showed a non-linear relationship between IMP and tissue hardness. The factors that most influenced the measurement were the age of the child, the place of measurement on the limb, which arm was dominant, and whether there was an active contraction of some of the muscles during measurement. An experimental device based on the same principle was tested on 5 patients and showed a correlation coefficient of 0.88 with IMP measurements (Molčányi, 2010).

In most of the studies performed, a correlation between tissue hardness and IMP was demonstrated. Measurements with this method are not possible continuously, but due to their non-invasive nature, they can be repeated very often. The factors that can skew the results include the amount of subcutaneous fat, age, and gender of the patient. However, no further study using this method has been published since 2011.

### Near-infrared spectroscopy

Near-infrared Spectroscopy (NIRS) is a method that uses electromagnetic radiation of the wavelengths at the near-infrared region to determine the state of oxygenation of hemoglobin. This method has been widely studied in recent years for monitoring oxygenation of the brain and muscles (Barstow, 2019). Tissue oxygenation decreases with decreasing tissue blood flow, which is lower in ECS due to lower CPP (Shuler et al., 2010). NIRS allows simple, non-invasive, and continuous measurement. The disadvantage is that the radiation penetrates only a few centimeters beneath the skin, so it does not penetrate, for example, into the deep posterior compartment of the lower leg (Scheeren et al., 2012).

Parameters that reduce the accuracy of the method include global reduction in tissue oxygenation and variability in the size and anatomy of the measured limbs. However, the measurements are most influenced by the concentrations of other substances that can absorb or reflect near-infrared radiation. The most common of these substances is skin pigment. The measurement can also be affected by the formation of a hematoma near the measurement site (Scheeren et al., 2012). When using this method, it is advisable to use a control measurement on the contralateral compartment. In this way, the error that can occur with a global lack of oxygen or increased skin pigment with a darker skin color can be avoided. When the limb is injured, hyperemia develops at the site of the injury, which increases the oxygen saturation values (Shuler et al., 2018).

When ECS develops, the regional tissue saturation (StO_2_) decreases. It is therefore important to continuously monitor changes in StO_2_ over time (Cole et al., 2014). Although bilateral injuries are common, it is not appropriate to use compartments in upper limbs in lower extremity injuries for control measurements. The most suitable place for control measurement is the contralateral compartment (Jackson et al., 2013).

The first studies to examine the suitability of NIRS for ECS detection were published at the beginning of the millennium when Garr et al. tested the correlation of NIRS results with IMP measurements and clinical signs in an animal model (Garr et al., 1999). The model was created by inflating a catheter balloon in the hind limbs of domesticated pigs. The concentration of oxyhemoglobin in the tissue was strongly inversely correlated with the measured IMP (r = −0.78). Budsberg et al. (Budsberg et al., 2016) used the same method of creating an animal model and their study confirms a strong inverse relationship between IMP and oxygenation value. This study further shows that increasing IMP increases tissue swelling and reduces CPP in the compartment.

The animal model was also used by Arbabi et al., But this time by infusion of albumin into the hind limb compartments of 9 pigs (Arbabi et al., 1999). This study examined whether a distinction could be made between muscular ischemia due to general hypoxia and muscular ischemia due to ECS. StO_2_ values in this study decreased from 82 ± 4% to 66 ± 10% due to general hypoxia, and ECS reduced these values further to 16 ± 12%.

Additional NIRS studies for ECS detection were performed on an ECS model in healthy volunteers by inflating a pressure cuff placed on the limb. When comparing NIRS to IMP on such a model, NIRS showed higher sensitivity than IMP (Gentilello et al., 2001). At a specificity value of 72% for both methods, the sensitivity of StO_2_ reached 94%. For IMP measurements, the sensitivity value was much lower, at 76%.

The NIRS method was also tested on patients with a clinically confirmed diagnosis of ECS (Giannotti et al., 2000). Compartments with ECS showed StO_2_ 56 ± 27% and IMP of 64 ± 17 mm Hg. before fasciotomy. After fasciotomy, IMP values returned to normal and StO_2_ increased to 82 ± 16%. Measurements on the deltoid of the same patients showed a mean tissue oxygenation value of 83%–87% over the whole duration of the measurement. The lower legs and deltoids of the patients in the control group were in the same range of StO_2_ values.

In a 2011 case study, Shuler et al. used a NIRS-based device INVOS (Somanetics Corp; Troy, Michigan USA) to monitor the development of compartment syndrome in 3 people (Shuler et al., 2011). In one case in this study, during 12 h of monitoring, the saturation value decreased from 80 to 58% at the time of fasciotomy in the deep posterior compartment of the leg, while remaining around 80% in the lateral compartment. This case points to the potential ability of NIRS to differentiate between ECS development in individual compartments.

The INVOS device was also used by another team in a case study with two patients with ECS (Aedo-Martín et al., 2019). The authors state that the NIRS method proved to be promising for the detection of ECS because, in both cases of measurement, the value of oxygenation increased rapidly after fasciotomy. For further testing it is necessary to use a much larger scale of testing with standardized parameters of measurement.

Schmidt et al. conducted an observational study of 191 patients with high-energy tibial injuries. IMP and blood pressure were measured continuously with an investigational device (Twin Star ECS Monitoring System; Minneapolis, Minnesota USA) and StO_2_ at the site of injury was measured with a Nonin Equanox 7600 (Nonin Medical; Plymouth, Minnesota USA). Only patients with one lower limb injury were included in the study to allow the other limb to be used as a control measurement site (Schmidt et al., 2017). Evaluation of data from these patients showed that only 31.6% of patients had clinically useful data from NIRS, whereas the IMP measurement method was able to provide usable data in 87.4% of cases. The authors explain the significant difference in the amount of successfully obtained data in two ways, by systematic errors in NIRS method data collection, which include both device-related errors and mistakes on the part of the medical staff, but they also question the ability of commercially available NIRS devices to measure oxygenation of injured limb tissue. Most studies do not deal with continuous data collection, but rather with shorter-term measurements. This study points to some limitations in continuous measurements. The reasons for these shortcomings may be bleeding, skin damage, or internal bleeding directly into the muscle compartment (Schmidt et al., 2018).

The problem with obtaining clinically useful data in continuous measurements is also reported by Shuler et al. in a clinical trial to validate NIRS as a method of detecting ECS (Shuler et al., 2018). In this study, NIRS was used in all 4 compartments of both lower limbs of 86 patients with one limb injury. The average oxygenation values of uninjured limbs ranged from 69% to 72%. In injured limbs, oxygenation was on average 3 % higher. All 7 limbs with confirmed ECS had oxygenation at least 3% lower in at least one compartment than in the uninjured compartments. The authors state that NIRS may be a suitable diagnostic modality in the diagnosis of ECS, but the method needs to be further validated by clinical trials and requires an increase in the reliability of data acquisition. Further clinical trials are currently underway, and detection of ECS using NIRS is by far the most studied method (Walters et al., 2019).

### Ultrasound

Diagnostic ultrasound is a widely used imaging method. Imaging of the muscle compartment does not reveal information about ECS development. The classic B mode ultrasound imaging of the muscle compartment was however used for measuring the tibiofascial angle, i.e., the angle between the anterolateral cortex of the tibia and the anterior compartment of the lower limb (Mühlbacher et al., 2019). According to the authors, the measured angle should increase during expansion and pressure increase in the anterior compartment. This hypothesis was tested on 40 cadaver limbs with simulated ECS. The study confirmed that the tibiofascial angle increased from 61° (±12.0°) at 10 mm Hg to 81° (±11.1°) at 100 mm Hg. This measurement is however only applicable to the anterior compartment of the lower limb and has not been verified on live subjects.

Imaging of a compartment at risk of developing ECS can however reveal hematomas that could cause ECS. Hematomas are not a prerequisite for ECS, but in specific cases, an ultrasound image may help make the diagnosis, as shown in two recent case studies (Eicken and Morrow, 2019; Long et al., 2021).

There are three methods that use ultrasound and may provide more information about the hardness of the tissues in the compartment or IMP itself. The first is a pulsed phase-locked loop (PPLL). This method takes advantage of the fact that peripheral pulse wave creates oscillations of the compartmental fascia. The amplitude of these oscillations is affected by IMP in the compartment (Lynch et al., 2009). The other two methods are shear wave elastography (SWE) and strain elastography (SE). Elastographic methods try to measure and quantify the hardness of tissues with the help of ultrasonic waves (Kim et al., 2018).

PPLL method uses an ultrasound probe equipped with a precise phase detector that allows the comparison of the phase shift of reflected waves against the phase of the transmitted waves. Phase shift is produced by slight changes in the frequency of the reflected wave caused by movement of the fascia toward or away from the probe. Using the PPLL method, it is possible to monitor small changes in the position of the targeted tissue in the order of micrometers. Monitoring the movements of compartment fascia during arterial pulsations is used for indirect measurement of IMP. It is supposed that at higher IMP, the amplitude of fascia displacements due to arterial pulsations increases. In compartment syndrome of the leg or forearm, monitoring of the interosseous membrane is used.

Based on this method, the EN-TACT device (Emergency Non-Invasive Tissue and Compartment Testing) was constructed. The device was manufactured by Luna Innovations, Inc. (Roanoke, Virginia USA). The device has not yet been approved for measuring IMP in clinical practice, but it has been used in research studies. A nonlinear dependence of the amplitude of fascia displacement on the increased IMP was found in a study on a model of ECS on 23 healthy individuals with an increased IMP by a cuff on the leg (Lynch et al., 2009). By recursively dividing the IMP values into 3 groups, where less than 30 mm Hg is normal pressure, 30 to 40 is marginal and greater than 40 is elevated, test sensitivity of 0.77 and specificity of 0.93 were achieved. It is not clear whether low sensitivity is due to errors in the creation of the ECS model, due to the use of cuffs, or due to the unsuitability of the method itself for IMP assessment.

A study on an animal model monitored IMP in sedated pigs with contralateral compartments as the control group (Garabekyan et al., 2009). The amplitude of the fascia displacements was monitored, and for each pressure increase, CPP was calculated. Subsequent analysis of the results showed that for CPP values from 80 to −40 mm Hg, the change in displacement amplitude differs significantly between tested compartments compared to controls. The fascial displacement in the monitored compartments at each change in CPP was significantly greater than in the control compartments (−20–40 mm Hg). The sensitivity of the method was determined to be 0.74 and the specificity to be 0.75. The results provide evidence for the correctness of the hypothesis that compartments with low CPP are characterized by a larger amplitude of fascia displacements during arterial pulsations. With increasing swelling and muscle hardness, the pressure transfer of arterial pulsations to the muscle fascia should theoretically change and thus the amplitude of its displacement should increase.

The advantage of this method compared to the classic IMP measurement is, in addition to the non-invasive nature of the method, also the possibility of continuous measurement of all 4 compartments. However, the results of the studies are limited due to the use of ECS models. In addition, the results show another important finding that although higher IMP increases the amplitude of fascia displacement, it reaches its highest values when approaching the mean arterial pressure. At higher values, it decreases again, because the blood flow begins to be interrupted at these pressure values.

A study by Lee et al. (2013), compared PPLL and NIRS methods along with IMP measurement with a slit catheter. Fifteen healthy volunteers were tested in whom the IMP was artificially increased using a pressure chamber. NIRS values were collected for each pressure value set by the chamber after finishing the measurement of PPLL. The results showed a statistically significant correlation between IMP and displacement amplitude in PPLL and between IMP and tissue oxygenation measured by NIRS. The cutoff value for compartment syndrome presence was taken to be 30 mm Hg of IMP. According to these criteria, the sensitivity and specificity for PPLL were 0.75 and for NIRS both values were 0.65. Thus, according to this study, the ultrasound PPLL method appears to be better for ECS detection. According to Wiemann et al. (2006), PPLL can detect increases in IMP and is therefore offered as a suitable method for the diagnosis of ECS.

The second category of the ECS methods using ultrasound is strain elastography that uses manual compression of tissues with an ultrasound probe while monitoring the pressure of the probe acting on the skin. In this way, it is possible to measure the qualitative elastogram of the examined tissue, where it is possible to discriminate between soft and hard tissues, because harder tissues show a lower degree of deformation when the area is compressed by the probe. In a way the measurement is like tissue hardness measurement mentioned earlier, but with the advantage of measuring the compartment dimensions that are affected by the compression force of the probe. The pressure sensor attachment on the probe is filled with water to minimize the effect on the measurement setup.

Sellei R. et al. (2015) used this method in several studies. In the pilot study, they used an ECS model (a container filled with water) and in the second study, they focused on human cadaver limbs (Sellei R. M. et al., 2015). These studies compared the depth of the compartment with no probe pressure with the depth at a probe pressure of 100 mm Hg. The difference in depth created by compressing the compartment was calculated. In both studies, this procedure was used for different values of artificially induced IMP from 0 to 80 mm Hg. Both studies showed a strong dependence of the measured depth difference on IMP. The results showed good repeatability of the measurements. However, the same results cannot be expected when applying the method to live patients, because the anisotropic properties of human muscles are likely to skew the measurement. ECS cannot be accurately modelled even in cadaver limbs.

A case study including six subjects showed promising results for detecting ECS when comparing relative elasticity of the anterior tibial muscle compartment with the contralateral compartment on the uninjured limb (Sellei R. M. et al., 2020). Relative elasticity of the compartment of less than 10.5% had a sensitivity of 95.8% and specificity of 87.5% for diagnosing ECS. The authors further studied this method and its inter and intra-observer repeatability in an *in vitro* model. Even though the repeatability of the measurements was high, the measurement process could be automatized to increase both the repeatability and simplicity of use for the method (Sellei R. et al., 2020).

Marmor et al. (2019) used strain elastography to analyze the anterior compartment on 6 human cadaver legs using ECS saline infusion model. The measured values of the compartment depth and values of the applied pressure required to change this depth were compared with the IMP values measured invasively with a Stryker pressure monitor. The results of the study show a high correlation of the measured parameters with IMP values. According to the authors, a higher correlation can be achieved when comparing an injured compartment to the control contralateral compartment. In this study, the authors introduced the CFFP marker (Compartment Fascia Flattening Pressure), i.e., the pressure that the probe must exert on the limb for the fascia of the compartment to change shape from convex to completely flat.

This coefficient was then used in another study (Herring et al., 2019), where the diagnostic value of this coefficient was verified on 10 patients with lower limb injuries without signs of ECS and 3 patients with a clinical diagnosis of ECS. The mean CFFP for the group of patients without ECS was 8 mm Hg, whereas, for patients with a confirmed diagnosis, the value of this coefficient was around 117 mm Hg. Bloch et al. (2018) in his study tested strain elastography in an animal model of ECS using 8 domesticated pigs infused with saline into the anterior tibial compartment. The measured parameter was the ratio of elasticity that is defined as the ratio of the depth of the compartment with the applied external pressure of the probe to the depth of the compartment without compression. IMP greater than or equal to 30 mm Hg was chosen as a criterion for the occurrence of ECS. At this pressure, the measured ratio of compartment elasticity reached 87.1% with sensitivity values of 94.4% and specificity of 88.9%.

The results of the available studies regarding strain elastography confirm this method could be a suitable non-invasive alternative for the detection of ECS. Compared to quantitative tissue hardness measurement, this method has the advantage of being able to view the entire compartment and ignore obstacles in the form of subcutaneous fat or hematomas. This method can also be used to monitor ECS development even in the deep posterior compartment. The advantage of this method is the use of commercially available devices, i.e., classic ultrasound linear probe in B-mode and a pressure sensor. However, despite good results, the accuracy and reliability of the method cannot be determined due to the small sample size and application on a model.

A difference between the SE and SWE (shear wave elastography) is that SE measures muscle hardness, which is defined as the resistance of muscle to pressure in a direction perpendicular to the muscle. SWE measures muscle stiffness that is the ratio of the change in force to the change in muscle length in the longitudinal axis (Inami and Kawakami, 2016). Shear wave elastography also produces a quantitative elastogram.

SWE has not yet been directly evaluated for ECS detection in a clinical setting, but there are studies suggesting that it may be suitable for this task. It is a relatively new imaging method that has already found many applications. The basic idea behind it is that pathologically altered tissues are usually stiffer than normal tissues. This method is routinely used in the diagnosis of breast cancer and is gradually more being used for other purposes. As the stiffness of the tissue increases, so does the speed of propagation of transverse ultrasound waves (Inami and Kawakami, 2016). This velocity is directly proportional to the shear modulus of the tissue, per equation:

$$\mu =\rho V_{s}^{2}$$ (1)

where µ indicates the shear modulus of the tissue, ρ the density of the tissue, and Vs. the velocity of propagation of the transverse ultrasound waves.

SWE was used to measure parameters of the anterior tibialis muscle at different angles of ankle flexion (Koo et al., 2014). The authors chose this compartment as their focus because it is the most frequent location of ECS. According to the authors, it could be possible to diagnose ECS non-invasively using this method. The results of the study show that the method can correctly measure the rate of propagation of transverse waves in the anterior tibialis muscle and their preliminary results from other measured muscles show that the transverse waves spread well in other muscles of the limbs as well, i.e., in the biceps brachii and brachioradialis. In certain muscles, the transverse waves may not propagate, for example in spinal erectors or trapezoids.

SWE has been investigated as a possible method for the diagnosis of carpal tunnel syndrome that is characterized by stiffness of the soft tissues of the wrist (Paluch et al., 2018). The study shows this method could be promising, but it was performed with small sample size and cannot yet be compared with more data.

When examining muscles with SWE, it is possible to observe even small changes in elasticity of the tissues that occur during muscle contractions. However, there are large differences in repeated measurements, both between measurements on the same patient and in absolute differences between different patients. Further research should address these shortcomings in repeatability with appropriate algorithms for better tolerance of the anisotropic properties of muscle tissue. However, the method has suitable properties that would be beneficial for the diagnosis of ECS (Creze et al., 2018).

A study looking at the relationship between IMP and the modulus of elasticity of muscles found a strong dependence of this modulus on IMP (Sadeghi et al., 2019a). Measurements were performed on 19 volunteers using a cuff to increase the pressure on the limb from 0 to 120 mm Hg. The IMP was measured using a catheter inserted into the muscle compartment. By increasing the IMP, a gradual increase in the measured value of tissue stiffness was also achieved. This relationship corresponded to a linear relationship with a correlation coefficient of 0.99, and the study, therefore, supports the possibility of using SWE to diagnose pathologies involving an increase in IMP.

A study looking at the possibility of using shear modulus to diagnose chronic compartment syndrome verified that the shear modulus increases during exercise and decreases back to baseline after 10 min of stopping exercise (Sadeghi et al., 2019b). The average value of the shear modulus increased from 14.12 kPa before exercise to 17.75 kPa 1 min after exercise. However, for further verification of the method, it is necessary to simultaneously measure IMP for comparison. A study on an animal model of ECS evaluating testicular compartment syndrome in rabbits after testicular torsion found a strong correlation between intratesticular pressure and testicular stiffness measured by shear wave elastography (Lin et al., 2020).

## Conclusion

Currently, physicians rely on diagnosis based solely on clinical symptoms. If the symptoms are not clear, invasive IMP measurement can be performed to aid in the diagnosis. Although this method has been proven to be reliable, it has several disadvantages. It cannot be used for long-term continuous monitoring; its invasive nature carries the risk of infection, and the measurement is painful for the patient. A simple non-invasive way of continuously monitoring the development of ECS would greatly enhance the quality of care for patients at risk. This work contains information about the studies that tried to find such a non-invasive method of measurement. None of the methods mentioned has been put into clinical practice yet.

The method that is closest to this is NIRS. It has shown promising results and is even the subject of clinical trials for this purpose. There are still technical limitations, not many NIRS devices are FDA approved, the current devices have shown limited reliability, and there are substantial differences in the obtained data across different NIRS devices (Shaaban-Ali et al., 2021). However, as NIRS has been extensively studied in the last years, it can be expected to become more standardized and available for clinical practice. It might not be the sole method used for making the diagnosis, but it can certainly aid in the decision-making process.

Probably the most promising of the methods investigated for ECS detection using ultrasound is the SE method that uses a pressure sensor attached to an ultrasound probe. It was recently tested on patients with tibial fractures, where the CFFP measurements correlated strongly with IMP measurements (Marmor et al., 2021). This method does not offer a continuous monitoring option, but it showed low inter and intra-observer variability. Using this method in a clinical setting with measurements in certain time intervals could prove valuable in monitoring the development of ECS. The biggest advantage of this method, when compared to SWE, is the price of equipment, SWE devices are more expensive than classic diagnostic ultrasound devices, where only a pressure sensor needs to be added. These two methods provide more information about the stiffness of the compartment than PPLL or simple ultrasound imaging. PPLL provides a continuous monitoring option but can be affected by variations in the blood pressure of the patient, which changes the amplitude of the fascia oscillations. Simple ultrasound imaging can reveal underlying problems in the compartment like hematomas but does not provide information about the overall stiffness of the compartment, so its use is limited.

It would be interesting to compare the ability of NIRS and SE methods for ECS detection in future studies. Based on the studies mentioned in this work, these two methods show the most promise and may find their way into clinical practice as a supplementary diagnostic tool that may even replace invasive IMP measurement as the gold standard in objective ECS development monitoring. This would be very beneficial for clinical practice because the measurement of IMP, like all invasive procedures, introduces certain risks that need to be considered.
